# Qualidade de Vida Relacionada à Saúde e Desfechos em Longo Prazo após COVID-19 Sintomática Leve: Protocolo do Estudo Pós-COVID Brasil 2

**DOI:** 10.36660/abc.20220835

**Published:** 2023-09-27

**Authors:** Marciane Maria Rover, Geraldine Trott, Fernando Luís Scolari, Mariana Motta Dias da Silva, Denise de Souza, Rosa da Rosa Minho dos Santos, Ana Paula Aquistapase Dagnino, Juliana de Mesquita, Gabriel Pozza Estivalete, Amanda Christina Kozesinski-Nakatani, Milena Soriano Marcolino, Bruna Brandão Barreto, Paulo Roberto Schvartzman, Ana Carolina Peçanha Antonio, Caroline Cabral Robinson, Maicon Falavigna, Andreia Biolo, Carisi Anne Polanczyk, Regis Goulart Rosa

**Affiliations:** 1 Hospital Moinhos de Vento Porto Alegre RS Brasil Projetos de Pesquisa – Hospital Moinhos de Vento, Porto Alegre, RS – Brasil; 2 Divisão de Cardiologia Hospital Moinhos de Vento Porto Alegre RS Brasil Divisão de Cardiologia – Hospital Moinhos de Vento, Porto Alegre, RS – Brasil; 3 Unidade de Terapia Intensiva Hospital Santa Casa de Curitiba Curitiba PR Brasil Unidade de Terapia Intensiva – Hospital Santa Casa de Curitiba, Curitiba, PR – Brasil; 4 Universidade Federal de Minas Gerais Belo Horizonte MG Brasil Medicina Interna – Universidade Federal de Minas Gerais, Belo Horizonte, MG – Brasil; 5 Departamento de Medicina Interna e Apoio Diagnóstico Faculdade de Medicina da Bahia Universidade Federal da Bahia Salvador BA Brasil Departamento de Medicina Interna e Apoio Diagnóstico – Faculdade de Medicina da Bahia – Universidade Federal da Bahia Salvador, BA – Brasil; 6 Unidade de Terapia Intensiva Hospital da Mulher – Maria Luzia Costa dos Santos Salvador BA Brasil Unidade de Terapia Intensiva – Hospital da Mulher – Maria Luzia Costa dos Santos, Salvador, BA – Brasil; 7 Unidade de Terapia Intensiva Hospital de Clínicas de Porto Alegre Porto Alegre RS Brasil Unidade de Terapia Intensiva – Hospital de Clínicas de Porto Alegre, Porto Alegre, RS – Brasil; 8 Unidade de Pesquisa Inova Medical Porto Alegre RS Brasil Unidade de Pesquisa – Inova Medical, Porto Alegre, RS – Brasil; 9 Instituto Nacional de Avaliação de Tecnologias em Saúde Universidade Federal do Rio Grande do Sul Porto Alegre RS Brasil Instituto Nacional de Avaliação de Tecnologias em Saúde – Universidade Federal do Rio Grande do Sul, Porto Alegre, RS – Brasil; 10 Faculdade de Medicina Universidade Federal do Rio Grande do Sul Porto Alegre RS Brasil Faculdade de Medicina – Universidade Federal do Rio Grande do Sul, Porto Alegre, RS – Brasil

**Keywords:** COVID-19, SARS-CoV-2, Sinais e Sintomas, Brasil

## Abstract

**Fundamento:**

Os efeitos em longo prazo da COVID-19 leve sobre a saúde física, mental e cognitiva ainda não são bem conhecidos.

**Objetivo:**

Este artigo visa descrever o protocolo para o estudo em andamento Pós-COVID Brasil 2, o qual tem como objetivo avaliar os fatores associados à qualidade de vida associada à saúde e desfechos cardiovasculares e não cardiovasculares de longo prazo um ano após um episódio de COVID-19 sintomática leve.

**Métodos:**

O estudo “Pós-COVID Brasil 2” é um estudo multicêntrico prospectivo que pretende incluir 1047 pacientes (NCT05197647). Entrevistas estruturas, centralizadas são conduzidas em um mês, e aos três, seis, nove e 12 meses após o diagnóstico de COVID-19. O desfecho primário é o escore de utilidade da qualidade de vida relacionada à saúde, avaliado usando o questionário EuroQol-5D-3L (EQ-5D-3L) aos 12 meses. Desfechos secundários incluem o EQ-5D-3L aos três, seis e nove meses, mortalidade por todas as causas, eventos cardiovasculares maiores, hospitalização, retorno ao trabalho ou à escola, sintomas persistentes, novas incapacidades em atividades instrumentais diárias, déficit cognitivo, ansiedade, depressão, e sintomas de transtorno do estresse pós-traumático as três, seis, nove e doze meses após a infecção pelo SARS-CoV-2. Um valor de p<0,05 será considerado estatisticamente significativo para as análises.

**Resultados:**

O desfecho primário será apresentado como frequência dos domínios do EQ-5D-3L doze meses após a infecção por SARS-CoV-2. A análise principal explorará a associação das variáveis independentes com os desfechos do estudo.

**Conclusão:**

O estudo “Pós-COVID Brasil 2” tem como objetivo elucidar o impacto da COVID longa sobre a qualidade de vida e desfechos cardiovasculares e não cardiovasculares de brasileiros pacientes que apresentaram COVID-19 leve.

## Figura Central

**Figure:**
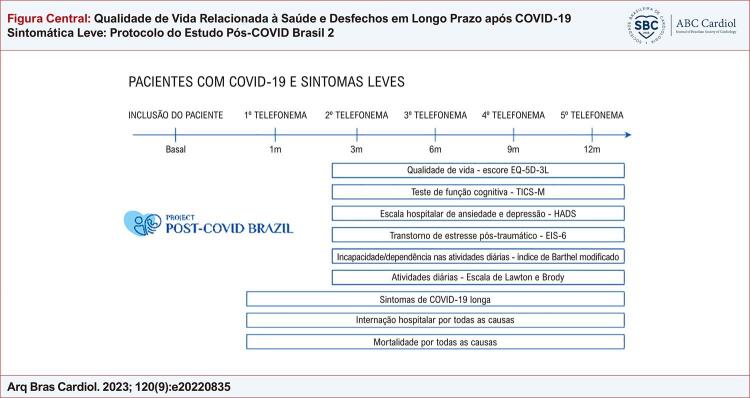
: Qualidade de Vida Relacionada à Saúde e Desfechos em Longo Prazo após COVID-19 Sintomática Leve: Protocolo do Estudo Pós-COVID Brasil 2

Delineamento do estudo Pós-COVID Brasil 2.

## Introdução

SARS-CoV-2, o coronavírus agente causador da COVID-19, infectou mais de 540 milhões de pessoas em todo o mundo, resultando em mais de seis milhões de mortes.^1,2^ No Brasil, mais de 34 milhões de casos de COVID-19 haviam sido relatados em novembro de 2022, sendo a maioria tratados ambulatorialmente.^1,3,4^ Sintomas persistentes após a infecção inicial pelo SARS-CoV-2 foram referidos como síndrome pós-COVID-19.^5^ Vale mencionar que a maioria dos pacientes com essa síndrome não tem uma história de doença grave ou internação hospitalar.^6-8^ De fato, um estudo mostrou que sintomas pós-COVID-19 eram frequentes, com 93% dos pacientes estudados (n=292) não conseguindo retornar ao estado de saúde basal após duas ou três semanas de um teste positivo para SARS-CoV-2.^9^ Os sintomas mais comumente relatados foram fadiga, dispneia, tosse, dor nas articulações, dificuldade de concentração, perda de memória, ansiedade, e depressão.^6-8^ A persistência dos sintomas seis meses após a infecção inicial foi associada com o número de comorbidades e carga do sintoma durante a fase aguda da COVID-19.^10^A Organização Mundial da Saúde (OMS) definiu recentemente a síndrome da COVID longa como sintomas que persistem ou se desenvolvem dentro de três meses de infecção,^11^ contanto que esses sintomas estejam presentes por pelo menos dois meses e não podem ser explicados por outras causas.

Eventos cardiovasculares foram relatados durante a fase aguda da COVID-19, mas dados recentes sugerem que insuficiência cardíaca, fibrilação atrial, pericardite, e outras condições cardíacas podem ocorrer em até 30 dias após a infecção aguda.^11,12^ Estudos mostraram que pelo menos 50% dos pacientes foram encaminhados para ambulatórios devido à dispneia por COVID longa, e 84% relataram pelo menos um sintoma cardiorrespiratório.^12^ Além disso, a incidência da insuficiência cardíaca aumentou em quase duas vezes entre pacientes com COVID-19 prévia dentro de nove meses da infecção aguda.^13^ Disfunção endotelial pós-COVID-19, avaliada por dilatação mediada por fluxo, também foi observada nesses pacientes, o que pode contribuir para o desenvolvimento da aterosclerose.^14,15^ Ainda, pacientes com doenças cardiovasculares crônicas estão em risco de descompensação em até 30 dias após a infecção aguda.^16^ Estudos mostraram que a elevação dos níveis de troponina durante a fase aguda da COVID-19 está associada com desfechos cardiovasculares maiores em longo prazo, destacando a lesão miocárdica causada pelo SARS-CoV-2.^17^

Estudos sobre a persistência de sintomas pós-COVID-19 incluíram primariamente pacientes com apresentação clínica moderada a grave, aqueles que necessitaram de internação, ou pacientes mais velhos com comorbidades.^18,19^ No entanto, a frequência de sintomas em longo prazo e o impacto da COVID-19 sobre a qualidade de vida de pacientes mais jovens com apresentação clínica leve são poucos conhecidos. Este é um protocolo do estudo Pós-COVID Brasil 2, que teve como objetivo avaliar os fatores associados à qualidade de vida relacionada à saúde (QVRS) um ano após a infecção leve por SARS-CoV-2. Os objetivos secundários foram avaliar a QVRS aos três, seis e nove meses, bem como mortalidade por todas as causas, eventos cardiovasculares, reinternação, retorno ao trabalho ou escola, sintomas persistentes, novas deficiências em atividades instrumentais da vida diária, déficit cognitivo, ansiedade, depressão e sintomas de estresse pós-traumático aos três, seis, nove e 12 meses após infecção leve por SARS-CoV-2.

## Métodos

**Figura 1:**
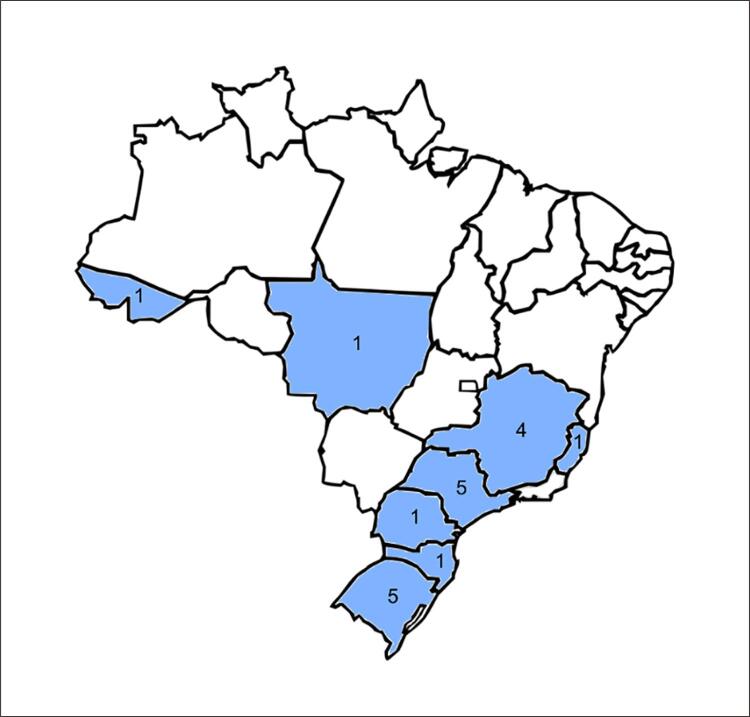
– Número de centros incluídos no Brasil por estado.

O estudo Pós-COVID Brasil 2 é delineado como um estudo prospectivo multicêntrico de pacientes que tiveram COVID-19 com sintomas leves e estão sendo tratados ambulatorialmente. Foram selecionados centros da maioria das regiões brasileiras (Figura 1) com capacidade de tratar pacientes com COVID-19. As exigências para a seleção estão descritas na Tabela 1. Os pacientes são convidados a participar do estudo ou pessoalmente, durante a consulta, ou por telefone após a visita ao ambulatório. O acompanhamento é conduzido por meio de entrevistas estruturadas, centralizadas, por telefone, por uma equipe de pesquisadores treinados em um mês, e aos três, seis, nove e 12 meses após o diagnóstico de COVID-19. O delineamento do estudo está ilustrado na Figura Central. O protocolo do estudo foi registrado no ClinicalTrials.gov (NCT05197647) antes da inclusão do primeiro participante. O estudo foi aprovado pelo comitê de ética institucional (protocolo 54665321.6.1001.5330) e segue a resolução número 466/12 do Conselho Nacional da Saúde. Consentimento informado por escrito ou eletrônico é obtido de cada participante no recrutamento.

**Tabela 1:** – Requerimentos para a seleção do centro

Centro de referência de casos de COVID-19, como uma sala de emergência, unidades de saúde, ou telemedicina;
Teste RT-PCR ou de antígeno para COVID-19 disponíveis;
Cumprimento do protocolo do estudo;
Consentimento por escrito para participação no estudo;
Aprovação do estudo por comitês de ética locais e autoridades éticas.

### Elegibilidade dos participantes

Pacientes com idade igual ou superior a 18 anos, com sintomas consistentes com infecção por SARS-CoV-2 e um teste positivo (RT-PCR ou antígeno) para COVID-19, são elegíveis para participação. Pacientes com doença subjacente e expectativa de vida de menos de três meses, determinado por avaliação clínica, pacientes sem suporte familiar, pacientes com deficiência na comunicação (afasia, deficiência cognitiva, falante de outra língua diferente da portuguesa), pacientes sem telefone, pacientes que retiraram seu consentimento, e aqueles incluídos no estudo anteriormente foram excluídos. Os critérios de elegibilidade para o estudo estão resumidos na Tabela 2.

**Tabela 2:** – Critérios de inclusão e exclusão do estudo Pós-COVID Brasil 2

Critérios de inclusão	
Idade ≥ 18 anos	
Teste RT-PCR positivo para SARS-CoV-2 com *swab* nasofaríngeo ou teste de antígeno positivo para SARS-CoV-2 com *swab* nasofaríngeo	
No mínimo um dos seguintes sintomas: Febre (> 38 °C), tosse, espirros, dispneia, perda ou alteração do olfato (anosmia) ou paladar (ageusia), coriza, dor de garganta, dor de cabeça, mialgia, dor na articulação, e diarreia	
Avaliação ambulatorial da infecção por SARS-CoV-2 sem necessidade de internação	
Critérios de exclusão	Racional
Expectativa de vida < três meses por comorbidade subjacente	A primeira entrevista de acompanhamento será realizada três meses após o diagnóstico de COVID-19.
Ausência de suporte familiar em um paciente com disfunção de linguagem ou de comunicação (afasia, déficit cognitivo, não ser falante da língua portuguesa)	A entrevista por telefone exige que o paciente se comunique com o entrevistador e entenda e responda as questões. Pacientes que não são capazes disso necessitarão de um membro da família
Ausência de número de telefone para contato	Todos os questionários serão administrados por entrevista telefônica.
Retirada do consentimento	A inclusão dos participantes sem consentimento resultaria em questões éticas
Participantes incluídos previamente no estudo	Dupla entrada no estudo levaria a um viés de seleção

## Desfechos

### Desfecho primário

O desfecho primário do estudo é o escore de utilidade de QVRS, medido usando o questionário EuroQol com cinco dimensões e três níveis (EQ-5D-3L) um ano após o diagnóstico de COVID-19.^20^ O EQ-5D-3L consiste em um sistema descritivo composto por cinco dimensões que descrevem a QVRS do paciente: mobilidade, autocuidado, atividades usuais, dor/desconforto e ansiedade/depressão. Cada dimensão tem três níveis de gravidade: nenhum problema, alguns problemas, e problemas extremos. Na população brasileira, o escore de utilidade derivou de um sistema descritivo que varia de -0,176 (representando o pior estado de saúde, com problemas graves em todas as dimensões) a 1 (indicando o melhor estado de saúde, sem nenhum problema).^20,21^ A mínima diferença clinicamente importante estimada para o EQ-5D-3L é 0,03, e o valor médio na população brasileira é 0,82.^22^ Os pacientes que forem a óbito durante o acompanhamento receberão um escore de zero nas avaliações restantes após o evento para assegurar que o impacto da morte sobre a qualidade de via é adequadamente refletida na análise.

### Desfechos secundários

Os desfechos secundários incluem vários parâmetros avaliados em diferentes momentos após a infecção pelo SARS-CoV2. Esses incluem o escore de utilidade EQ-5D-3L avaliado em um mês, e aos três, seis e nove meses após a infecção, mortalidade por todas as causas, eventos cardiovasculares maiores (morte cardiovascular, infarto agudo do miocárdio não fatal, e acidente vascular cerebral não fatal, avaliados individualmente e combinados), internação hospitalar, e sintomas persistentes de COVID-19 (como dispneia, tosse, fadiga, fraqueza muscular, dor torácica, dor nas articulações, alteração no olfato ou no paladar, queda de cabelo, dificuldade de se concentrar, e distúrbios do sono). Ainda, a disfunção cognitiva é avaliada pela entrevista telefônica modificada para avaliação do estado cognitivo (TICS-M, *Telephone Interview for Cognitive Status-modified*),^23,24^ sintomas de ansiedade e depressão estimados pela escala HADS (*Hospital Anxiety and Depression Scale*),^25,26^ e transtorno de estresse pós-traumático é avaliado usando a escala de impacto do evento IES-6 (*Impact of Event Scale-6*) aos três, seis, nove e doze meses.^27,28^ O estado físico funcional é avaliado usando o índice de Barthel modificado,^29^ e novas deficiências em atividades instrumentais diárias são avaliadas usando a escala de Lawton & Brody, que avalia qualquer deficiência em domínios como uso do telefone, transporte, compras, responsabilidade por seus medicamentos, e capacidade em lidar com finanças.^30,31^ Os desfechos secundários também incluem o retorno ao trabalho ou à escola, e reinfecção sintomática pelo SARS-CoV-2 (definida como recorrência de sintomas de COVID-19 e infecção confirmada por RT-PCR ou antígeno positivo para SARS-CoV-2 mais de 90 dias após a infecção primária), avaliados três, seis, nove, e 12 meses após a infecção por SARS-CoV-2 e descritos como frequência e incidência.

### Fatores associados ou variáveis

O estudo avaliará cinco conjuntos de variáveis quanto a potenciais associações prognósticas. O primeiro conjunto inclui variáveis demográficas tais como sexo, idade, educação, e renda familiar média. O segundo conjunto inclui comorbidades tais como doenças cardíacas (por exemplo, angina, infarto agudo do miocárdio, e insuficiência cardíaca), doenças cerebrovasculares (como acidente vascular cerebral e ataque isquêmico transitório), demência, doença vascular periférica, doença pulmonar obstrutiva crônica, diabetes, doença do tecido conjuntivo, doença hepática, doença renal crônica, tumores sólidos, leucemia, linfoma, mieloma, AIDS, receptores de transplante de órgãos sólidos, transplante de medula óssea, hipertensão pulmonar, terapia imunossupressora, e distúrbios de humor, entre outros. O índice de comorbidade de Charlson será calculado para esse conjunto de variáveis. O terceiro conjunto de variáveis é o status de vacinação. O quarto conjunto avalia a gravidade da COVID-19 na apresentação inicial de acordo com a classificação da Organização Mundial da Saúde, que inclui pacientes não hospitalizados, com e sem disfunção funcional. O quinto conjunto avalia exames laboratoriais e de imagem complementares na apresentação da doença, incluindo proteína C reativa, dímeros D, troponina, peptídeo natriurético cerebral, contagem de linfócitos, tomografia computadorizada do tórax, e radiografia do tórax.

### Acompanhamento

O período de acompanhamento inicia-se no dia em que o participante assina o termo de consentimento (data de referência para as entrevistas). A equipe de pesquisadores do hospital Moinhos de Vento, que são treinados para coletarem dados e conduzirem as chamadas telefônicas de seguimento, contatam todos os participantes um, três, seis, nove e doze meses após o acompanhamento inicial. Todas as chamadas são realizadas dentro de um período de mais ou menos 15 dias antes ou após a data esperada do telefonema, e registradas em um banco de dados eletrônico. Perda no seguimento por telefone é definido como ausência de contato após 10 tentativas consecutivas em dias diferentes e mudança do número telefônico. Haverá perda do seguimento se ocorrer problema de conexão em duas ocasiões consecutivas ou se houver um erro em um dos números que não possa ser resolvido. Durante a entrevista, os indivíduos incluídos respondem perguntas de um questionário estruturado que aborda estado de vida, história de internação, retorno ao trabalho ou à escola, o questionário EQ-5D-3L, persistência dos sintomas, e todos os desfechos anteriormente mencionados. Um membro da família ou representante legal pode responder todas as perguntas, exceto aquelas relacionadas aos instrumentos HADS, IES-6, e TICS-m. Dados do EQ-5D-3L, da escala analógica visual e da escala de Lawton e Brody um mês antes do diagnóstico da COVID-19, são coletados retrospectivamente durante os telefonemas de acompanhamento. Se informações sobre mortalidade por todas as causas, eventos cardiovasculares maiores (morte cardiovascular, infarto agudo do miocárdio não fatal, e acidente vascular cerebral não fatal), internação dentro de 12 meses da entrada no estudo, ou retorno ao trabalho ou à escola estiverem faltando, elas podem ser avaliadas retrospectivamente em qualquer telefonema subsequente.

### Processamento para qualidade dos dados

Formulários *online* padrões para relato de casos, disponíveis para smartphones, tablets, e computadores são usados para registro dos dados. A coleta e o manejo dos dados *online* têm vários benefícios incluindo a padronização, a confiabilidade e a segurança dos dados. Todos os dados são armazenados e gerenciados no REDCap (Research Electronic Data Capture – https://www.redcapbrasil.com.br).^32^ O pesquisador principal atribui um nome de usuário e uma senha únicos e intransferíveis a cada pesquisador para acessar a plataforma do estudo. Há, também, permissões específicas dentro da plataforma.

### Qualidade e segurança dos dados

Para assegurar a qualidade e a segurança dos dados, os seguintes procedimentos são seguidos:^33-35^ 1. Toda a equipe de pesquisa passa por um treinamento sobre boas práticas clínicas, procedimentos do estudo, e coleta de dados; 2. Todos os pesquisadores têm acesso ao centro coordenador para resolver problemas relacionados ao estudo; 3. Todo o gerenciamento dos dados está de acordo com a lei 13.709 geral de proteção de dados de 14 de agosto de 2018;^36^ 4. O acesso ao banco de dados é protegido por um nome de usuário e uma senha únicos e intransferíveis fornecidos a cada participante do estudo; 5. Uma cópia de segurança dos dados é automaticamente feita a cada 24 horas. A extração dos dados para análise estatística é realizada com anonimização de dados, permitindo a verificação da consistência dos dados e monitoramento remoto dos processos; 6. O pesquisador principal verifica periodicamente a consistência dos dados. Em caso de erro, os pesquisadores são notificados e solicitados a corrigir a entrada dos dados; 7. As chamadas telefônicas são gravadas e auditadas para consistência dos dados. Os arquivos dos áudios são armazenados com anonimização dos dados em um servidor similar àquele usado para os dados clínicos. O acesso aos arquivos de áudio é permitido por meio de um nome de usuário e uma senha; 8. O centro coordenador revisa, mensalmente, os relatórios detalhados sobre o rastreamento, critérios de inclusão, e acompanhamento dos pacientes, consistência dos dados, e completude dos dados e imediatamente toma medidas para resolver qualquer problema; e 9. Procedimentos estatísticos são realizados ao longo do estudo para identificar potenciais fraudes.

### Tamanho amostral

Um tamanho amostral estimado de 906 pacientes não hospitalizados com COVID-19 é necessário para realizar uma regressão linear múltipla com cinco preditores e uma correlação cruzada de 0,25 de acordo com o método PEAR.^33^ Considerando uma taxa de 10% de perda de seguimento ou retirada de consentimento, a amostra final incluirá 997 participantes. Devido a uma taxa de internação antecipada de 2-5% em 21 dias, o tamanho da amostra aumentará para 1047 na primeira análise dos pacientes não internados.

### Análise estatística

A análise estatística será realizada uma vez que forem conduzidos a obtenção de todos os dados, a limpeza e o bloqueio do conjunto de dados, e a submissão do protocolo para publicação. Primeiramente, será conduzida uma avaliação descritiva dos dados. As variáveis categóricas serão apresentadas em frequência relativa e absoluta. As variáveis contínuas serão inicialmente avaliadas quanto à distribuição usando o teste de Shapiro-Wilk e inspeção visual dos histogramas das variáveis. As variáveis contínuas serão apresentadas como média e desvio padrão ou mediana e intervalos interquartis. A associação entre os desfechos do estudo e as variáveis independentes será analisada usando Equações de Estimativa Generalizada (EEG), tanto univariadas como multivariadas. Serão apresentados os valores ajustados e os valores não ajustados, bem como os intervalos de confiança de 95% e valores p para cada estimativa. Serão realizadas análises exploratórias de subgrupos quanto aos desfechos. Para as variáveis contínuas, será realizado o teste t ou o teste de Wilcoxon, dependendo da distribuição dos dados, e para as variáveis categóricas, será usado o teste do qui-quadrado. Modelos de regressão também serão conduzidos, dependendo do tipo do desfecho de interesse. Um nível de significância de 0,05 e um intervalo de confiança de 95% serão considerados para todas as análises estatísticas.

O centro coordenador entrará em contato com pesquisadores locais para corrigir dados inconsistentes ou faltantes. Se os dados continuarem faltantes, os dados basais não serão substituídos. Avaliações do EQ-5D-3L faltantes serão substituídas pela última avaliação realizada, exceto para pacientes que foram a óbito, que receberão um escore de zero em toda o acompanhamento após o evento. Valores faltantes para o índice de Barthel, HADS e IES-6 serão substituídos pela média dos itens respondidos na mesma subescala, se pelo menos metade da subescala tiver sido respondida. As análises estatísticas serão realizadas usando o programa R, versão 4.2.2 (R Foundation for Statistical Computing).

### Aspectos éticos e de disseminação

#### Aprovação ética e consentimento

O estudo está de acordo com as diretrizes descritas na resolução 466 de 12 de dezembro de 2012 do Conselho Nacional da Saúde, com o adendo E6 das diretrizes de boas práticas clínicas (segunda revisão) do *International Council for Harmonization*, e com a lei 13.709 geral de proteção de dados de 14 de agosto de 2018. O estudo foi iniciado somente após a aprovação do protocolo pelos comitês de ética das instituições. Consentimento por escrito ou eletrônico é obtido de cada participante elegível no momento do recrutamento, e a linguagem utilizada é clara e inclusiva, ao fornecer informações sobre objetivos, metodologia, coleta de dados, e processo de registro do estudo, de acordo com a resolução 466/2012 do Conselho Nacional de Saúde. O pesquisador local lê o termo de consentimento aos participantes rastreados ou seus representantes legais, explicando os potenciais riscos e benefícios do estudo. Todos os participantes são voluntários e podem retirar o consentimento em qualquer momento, sem impacto sobre seu tratamento. O pesquisador também informa aos participantes que seu registro de identificação será gravado e poderá ser acessado pelas autoridades locais de vigilância sanitária e pelo centro coordenador, sem violar a confidencialidade dos participantes. O termo de consentimento é registrado com a data atual e assinado tanto pelo participante (ou representante legal) como pelo pesquisador somente após o esclarecimento dos procedimentos do estudo e antes da aplicação de qualquer protocolo. Uma cópia do termo de consentimento fica com o participante, e a outra com o pesquisador.

De acordo com a Carta Circular número 2/2021 da Comissão Nacional de Ética em Pesquisa, vinculada ao Ministério da Saúde, emitida em 24 de fevereiro de 2021, que dá orientações para procedimentos em pesquisas e atividades de comitês de éticas em ambiente virtual durante a pandemia da COVID-19, uma assinatura digital pode ser aceita em centros em que pacientes são acompanhados remotamente ou quando os pacientes ou seus representantes legais não conseguem ir ao centro. Independentemente do formato, o pesquisador é responsável por confirmar o consentimento.

#### Disseminação

Os pesquisadores pretendem apresentar os resultados do estudo em reuniões médicas e congressos, e preparar o artigo para publicação em uma revista médica com revisão por pares. O comitê de direção do estudo determinará quais resultados serão publicados e a que revista eles serão submetidos. A autoria será determinada seguindo-se a definição do *International Committee of Medical Journal Editors* (ICMJE).

## Compartilhamento de dados

Os autores encorajam pesquisadores terceiros a contatarem o autor de correspondência para compartilhar os dados e a acessarem os dados não publicados. O uso de um aplicativo para compartilhamento de dados está sob consideração pelo comitê de direção do estudo.

## Discussão e atualização do estudo

Nosso estudo tem como objetivo investigar o impacto da COVID-19 longa sobre a qualidade de vida e desfechos de pacientes que tiveram COVID-19 leve. A maioria dos estudos anteriores sobre persistência dos sintomas focaram em pacientes com COVID-19 moderada a grave, que necessitaram de internação e apresentaram alta carga de comorbidades.^6-8^ Com o foco nos pacientes com COVID-19, nosso estudo pode identificar aqueles com risco de desenvolverem sintomas de longo prazo, e divulgar políticas públicas de saúde que visem subgrupos específicos de pacientes com maior probabilidade de se beneficiarem de um acompanhamento.

Uma revisão sistemática que analisou nove estudos de pacientes com COVID-19 leve e um acompanhamento de várias semanas após a infeção mostrou que a persistência de sintomas além de três semanas variou entre 10% e 35%, sendo a fadiga o sintoma mais comum. Outros sintomas persistentes após a infecção incluem dispneia, tosse, dor torácica, cefaleia, declínio mental e cognitivo, e distúrbios de paladar e do olfato. Ainda, a persistência dos sintomas após a infecção por SARS-CoV-2 tem um impacto significativo sobre as atividades relacionadas ao trabalho.^37^ Contudo, o impacto dos sintomas com duração acima de três meses continua desconhecido.

Dados populacionais do Brasil durante o primeiro ano da pandemia mostraram um aumento na mortalidade cardiovascular.^38^ Isso é preocupante considerando que o vírus SARS-CoV-2 pode ter um impacto sobre o sistema cardiovascular por vários mecanismos, incluindo disfunção microvascular, disparidade entre o fornecimento e a demanda de oxigênio, lesão miocárdica direta, e toxicidade no cardiomiócitos durante a fase aguda da doença.^14,39^Esses mecanismos podem também contribuir para desfechos cardiovasculares em longo prazo. Portanto, é importante avaliar e acompanhar pacientes quanto às sequelas cardiovasculares para identificar aqueles em risco e tratar doenças cardíacas secundárias como a insuficiência cardíaca.^13,16,40^ Apesar desses esforços, a carga real da COVID longa sobre a doença cardiovascular continua desconhecida e pode demandar mais tempo para ser determinada. O estudo Pós-COVID Brasil 2 tem como objetivo elucidar não só a carga dos sintomas cardiovasculares em pacientes com COVID-19 bem como seus desfechos em longo prazo.

Os pontos fortes deste estudo são seu delineamento prospectivo, que inclui um grande número de pacientes com COVID-19 leve de vários centros no Brasil, e avaliação centralizada dos desfechos, com um seguimento de 12 meses. No entanto, potenciais limitações incluem interpretação subjetiva dos sintomas após a COVID-19, que pode ser influenciada por consultas médicas e testes complementares conduzidos durante o acompanhamento. Essa abordagem de autoavaliação torna difícil determinar se os sintomas resultam da infecção por SARS-CoV2 ou de comorbidades subjacentes.

O delineamento e o protocolo do estudo foram concluídos em dezembro de 2021. O recrutamento dos pacientes era esperado de ser completado em dezembro de 2022, mas o rastreamento continuará até a população alvo ser alcançada. Na época em que este artigo foi escrito, um terço do número total de participantes haviam sido incluídos no estudo, com 27 centros de recrutamento ativos. Os telefonemas aos participantes iniciaram-se em 24 de fevereiro de 2022. Os autores esperam completar o acompanhamento de um ano da população em dezembro de 2023.
