# Supplementary material for: Qualidade de Vida Relacionada à Saúde e Desfechos em Longo Prazo após COVID-19 Sintomática Leve: Protocolo do Estudo Pós-COVID Brasil 2
Source: Arq Bras Cardiol. 2023 Sep 27;120(9):e20220835. [Article in Portuguese] doi: 10.36660/abc.20220835 (PMC10547435; doi:10.36660/abc.20220835)
Supplement: Translation

# Translation: Health-Related Quality of Life and Long-Term Outcomes after Mildly Symptomatic COVID-19: The Post-COVID Brazil Study 2 Protocol

**Authors:** Marciane Maria Rover, Geraldine Trott, Fernando Luís Scolari, Mariana Motta Dias da Silva, Denise de Souza, Rosa da Rosa Minho dos Santos, Ana Paula Aquistapase Dagnino, Juliana de Mesquita, Gabriel Pozza Estivalete, Amanda Christina Kozesinski-Nakatani, Milena Soriano Marcolino, Bruna Brandão Barreto, Paulo Roberto Schvartzman, Ana Carolina Peçanha Antonio, Caroline Cabral Robinson, Maicon Falavigna, Andreia Biolo, Carisi Anne Polanczyk, Regis Goulart Rosa

**Affiliations:** 1 Brasil Projetos de Pesquisa – Hospital Moinhos de Vento, Porto Alegre, RS – Brasil; 2 Brasil Divisão de Cardiologia – Hospital Moinhos de Vento, Porto Alegre, RS – Brasil; 3 Brasil Unidade de Terapia Intensiva – Hospital Santa Casa de Curitiba, Curitiba, PR – Brasil; 4 Brasil Medicina Interna – Universidade Federal de Minas Gerais, Belo Horizonte, MG – Brasil; 5 Brasil Departamento de Medicina Interna e Apoio Diagnóstico – Faculdade de Medicina da Bahia – Universidade Federal da Bahia Salvador, BA – Brasil; 6 Brasil Unidade de Terapia Intensiva – Hospital da Mulher – Maria Luzia Costa dos Santos, Salvador, BA – Brasil; 7 Brasil Unidade de Terapia Intensiva – Hospital de Clínicas de Porto Alegre, Porto Alegre, RS – Brasil; 8 Brasil Unidade de Pesquisa – Inova Medical, Porto Alegre, RS – Brasil; 9 Brasil Instituto Nacional de Avaliação de Tecnologias em Saúde – Universidade Federal do Rio Grande do Sul, Porto Alegre, RS – Brasil; 10 Brasil Faculdade de Medicina – Universidade Federal do Rio Grande do Sul, Porto Alegre, RS – Brasil

**Keywords:** COVID-19, SARS-CoV-2, Signs and Symptoms, Brazil

## Abstract

**Background:**

The long-term effects of mild COVID-19 on physical, cognitive, and mental health are not yet well understood.

**Objective:**

The purpose of this paper is to describe the protocol for the ongoing “Post-COVID Brazil” study 2, which aims to evaluate the factors associated with health-related quality of life and long-term cardiovascular and non-cardiovascular outcomes one year after a mild episode of symptomatic COVID-19.

**Methods:**

The “Post-COVID Brazil” study 2 is a prospective multicenter study that plans to enroll 1047 patients (NCT05197647). Centralized, structured telephone interviews are conducted at 1, 3, 6, 9, and 12 months after COVID-19 diagnosis. The primary outcome is the health-related quality-of-life utility score, assessed using the EuroQol-5D-3L (EQ-5D-3L) questionnaire at 12 months. Secondary endpoints include the EQ-5D-3L at 3, 6, and 9 months, as well as all-cause mortality, major cardiovascular events, hospitalization, return to work or education, persistent symptoms, new disabilities in instrumental activities of daily living, cognitive impairment, anxiety, depression, and post-traumatic stress symptoms at 3, 6, 9, and 12 months after SARS-CoV-2 infection. A p-value < 0.05 will be considered statistically significant for all analyses.

**Results:**

The primary endpoint will be presented as the overall frequency of the EQ-5D-3L domains 12 months after SARS-CoV-2 infection. Main analysis will explore the association of independent variables with the study outcomes.

**Conclusion:**

The “Post-COVID Brazil” study 2 aims to clarify the impact of long COVID on the quality of life and cardiovascular and non-cardiovascular outcomes of Brazilian patients who have had mild COVID-19.

## Introduction

SARS-CoV-2, the coronavirus that is the causative agent of COVID-19, has infected over 540 million people worldwide, resulting in more than six million deaths.^1,2^ In Brazil, more than 34 million COVID-19 cases were reported in November 2022, with most cases treated as outpatients.^1,3,4^ Persistent symptoms following initial SARS-CoV-2 infection have been referred to as post-acute COVID-19 syndrome.^5^ It is worth noting that most patients with this syndrome do not have a history of severe disease or hospitalization.^6-8^ In fact, a study showed that post-COVID-19 symptoms were common, with 93% of the study patients (n=292) failing to return to their baseline state of health even after two to three weeks of a positive SARS-CoV-2 test.^9^ The most commonly reported symptoms were fatigue, dyspnea, cough, joint pain, difficulty concentrating, memory loss, anxiety, and depression.^6-8^ Persistence of symptoms six months after the initial infection was associated with the number of comorbidities and symptom burden during the acute phase of COVID-19.^10^ The World Health Organization (WHO) has recently defined long-COVID syndrome as symptoms that persist or develop within three months of infection,^11^ provided that the symptoms are present for at least two months and cannot be explained by other causes.

Cardiovascular events have been reported during the acute phase of COVID-19, but recent data suggest that heart failure, atrial fibrillation, pericarditis, and other cardiac conditions may occur up to 30 days after the acute infection.^11,12^ Studies have shown that at least 50% of patients were referred to outpatient clinics due to long COVID dyspnea, while 84% reported at least one cardiopulmonary symptom.^12^Furthermore, the incidence of heart failure has increased by almost two-fold among patients with previous COVID-19 within nine months of the acute infection.^13^ Post-COVID-19 endothelial dysfunction, assessed by flow-mediated dilation, has also been observed in these patients, which may contribute to atherosclerosis.^14,15^ Additionally, patients with chronic cardiovascular conditions are at risk of decompensation up to 30 days after the acute infection.^16^ Studies have shown that troponin elevation during the acute phase of COVID-19 is associated with major long-term cardiovascular outcomes, underscoring the myocardial injury caused by SARS-CoV-2.^17^

Studies on post-COVID-19 symptom persistence have primarily included patients with moderate-to-severe clinical presentation, those requiring hospitalization, or older patients with comorbidities.^18,19^ However, the frequency of long-term symptoms and the impact of COVID-19 on quality of life in younger patients with mild clinical presentation are still poorly understood. This is a protocol of the Post-COVID Brazil study 2 that sought to evaluate the factors associated with health-related quality of life (HRQOL) one year after mild SARS-CoV-2 infection. The secondary objectives are to evaluate HRQOL at three, six, and nine months, as well as all-cause mortality, cardiovascular events, rehospitalization, return to work or education, persistent symptoms, new disabilities in instrumental activities of daily living, cognitive impairment, and anxiety, depression, and post-traumatic stress symptoms at 3, 6, 9, and 12 months after mild SARS-CoV-2 infection.

## Methods

The post-COVID Brazil study 2 is designed as a prospective multicenter study of patients who have had COVID-19 with mild symptoms and are being treated as outpatients. Centers across most regions of Brazil (as shown in Figure 1) that have the capacity to manage patients with COVID-19 have been selected. The requirements for center selection are outlined in Table 1. Patients are enrolled in person during clinical evaluation or by telephone after visiting the healthcare facility. Follow-up is entirely conducted through centralized, structured telephone interviews that are performed by a team of trained researchers at one, three, six, nine, and 12 months after COVID-19 diagnosis. The study design is illustrated in the central illustration. The study protocol has been registered on ClinicalTrials.gov (NCT05197647) prior to the recruitment of the first participant. The study has been approved by the Institutional Ethics Committee (approval number, 54665321.6.1001.5330) and meets the Brazilian National Health Council Resolution 466/12. Written or electronic informed consent is obtained from each participant at the time of enrollment.

**Figure 1:** – Number of included centers by federal states in Brazil.

Post-COVID Brazil study 2 design.

**Table 1:** – Requirements for center selection

Referral health center for evaluation of COVID-19 cases as an emergency room, health centers or telehealth
COVID-19 RT-PCR test or antigen test available;
Compliance with the study protocol;
Written consent to participate in the study;
Study approval by the local research ethics committee and ethical authorities.

### Patient eligibility

Patients aged 18 years or older with clinical symptoms consistent with SARS-CoV-2 infection and a positive COVID-19 RT-PCR or antigen test are eligible for participation. Patients with an underlying illness and a life expectancy of less than three months, as determined by clinical judgement, patients without family support, who have communication impairment (aphasia, cognitive deficit, non-Portuguese speaker), patients without a telephone, patients who have withdrawn their consent, and those previously included in the study are excluded. The eligibility criteria for the study are summarized in Table 2.

**Table 2:** – Inclusion and exclusion criteria of the post-COVID Brazil study 2

Inclusion criteria	
≥ 18 years of age	
RT-PCR test positive for SARS-CoV-2 with nasopharyngeal swab or antigen test positive for SARS-CoV-2 with nasopharyngeal swab	
At least one of the following symptoms: Fever (> 38 °C), cough, sneezing, dyspnea, loss or impairment of the sense of smell (anosmia) or taste (ageusia), coryza, sore throat, headache, myalgia, joint pain, and diarrhea	
Outpatient evaluation for SARS-CoV-2 infection without need for hospitalization	
Exclusion criteria	Rationale
Life expectancy < 3 months due to underlying comorbidity	The first follow-up interview will be held 3 months after the COVID-19 diagnosis.
Absence of familial support in a patient with language/communication impairment (aphasia, cognitive deficit, non-Portuguese speaker)	The telephone interview requires patients to communicate with the interviewer and to understand and answer the questions. Patients unable to do so would require a family member.
No telephone available for contact	All questionnaires will be administered via telephone interview.
Withdrawal of consent	The inclusion of participants without consent would result in ethical issues.
Participants previously included in the study	Double entry in the study would lead to selection bias.

## Outcomes

### Primary outcome

The primary endpoint of the study is the HRQOL utility score, measured using the EuroQol 5-dimension 3-level (EQ-5D-3L) questionnaire at one year after the COVID-19 diagnosis.^20^ The EQ-5D-3L questionnaire consists of a descriptive system comprising five dimensions that describe the patient’s HRQOL: mobility, self-care, usual activities, pain/discomfort, and anxiety/depression. Each dimension has three levels of severity: no problems, some problems, and extreme problems. In the Brazilian population, the utility score derived from the descriptive system ranges from -0.176 (representing the worst health state, with severe problems in all dimensions) to 1 (indicating the best health state, with no problems at all).^20,21^ The estimated minimal clinically important difference for the EQ-5D-3L is 0.03, and the mean value in the Brazilian population is 0.82.^22^ Patients who die during the follow-up period will receive a score of zero in the remaining assessments after the event to ensure that the impact of death on quality of life is adequately reflected in the analysis.

### Secondary outcomes

Secondary outcomes include multiple parameters assessed at various time points after SARS-CoV-2 infection. These include the EQ-5D-3L utility score at one, three, six, and nine months after infection, all-cause mortality, major cardiovascular events (cardiovascular death, non-fatal acute myocardial infarction, and non-fatal stroke, evaluated individually and combined), hospitalization, and persistent COVID-19 symptoms (e.g., dyspnea, cough, fatigue, muscle weakness, chest pain, joint pain, smell or taste impairment, hair loss, difficulty concentrating, and sleep disorders). Additionally, cognitive impairment is assessed using the Telephone Interview for Cognitive Status-modified (TICS-m),^23,24^ anxiety and depression symptoms are estimated by the Hospital Anxiety and Depression Scale (HADS),^25,26^ and post-traumatic stress disorder is evaluated using the Impact of Event Scale-6 (IES-6) at three, six, nine, and 12 months.^27,28^ Functional physical status is assessed using the modified Barthel index,^29^ and new disabilities in instrumental activities of daily living are evaluated using the Lawton & Brody instrumental activities of daily living scale, which assesses any impairment in domains such as telephone use, transportation, shopping, responsibility for own medications, and ability to handle finances.^30,31^ Secondary outcomes also include return to work or education, and symptomatic SARS-CoV-2 reinfection (defined as the recurrence of COVID-19-like symptoms and infection confirmed by an RT-PCR or antigen test positive for SARS-CoV-2 more than 90 days after primary infection), assessed at 3, 6, 9, and 12 months after SARS-CoV-2 infection and reported as frequency and incidence.

### Associated factors or prognostic variables

The study will evaluate five sets of variables for potential prognostic associations. The first set includes demographic variables such as age, sex, education, and mean family income. The second set includes comorbidities such as cardiac diseases (e.g., angina, acute myocardial infarction, and heart failure), cerebrovascular diseases (e.g., stroke and transient ischemic attack), dementia, peripheral vascular disease, chronic obstructive pulmonary disease, diabetes, connective tissue disease, hepatic disease, chronic kidney disease, solid tumors, leukemia, lymphoma, myeloma, AIDS, solid organ transplant recipients, bone marrow transplant, pulmonary artery hypertension, immunosuppressive therapy, and mood disorders, among others. The Charlson Comorbidity Index will be calculated for this set of variables. The third set of variables is COVID-19 vaccination status. The fourth set evaluates the severity of COVID-19 at initial presentation according to the WHO classification, which includes non-hospitalized patients with and without functional impairment. The fifth set evaluates complementary laboratory and imaging tests at disease presentation, including C-reactive protein, D-dimers, troponin, brain natriuretic peptide, lymphocyte count, chest computed tomography, and chest X-ray.

### Follow-up

The follow-up period begins on the day the participant signs the consent form (reference date for the interviews). The Hospital Moinhos de Vento research team members, who are trained to collect data and conduct follow-up telephone calls, contact all participants at 1, 3, 6, 9, and 12 months after the initial follow-up. All calls are made within a 15-day period range of the expected call date and are recorded in an electronic database. A loss to telephone follow-up is defined as no contact after 10 consecutive attempts on different days and change of phone number. Loss to follow-up will also occur if there is a connection problem on two consecutive occasions or if there is a typo in the telephone number that cannot be resolved. During the interview, enrolled individuals are asked questions from a structured questionnaire that address their vital status, hospitalization history, return to work or education, the EQ-5D-3L questionnaire, symptom persistence, and all the previously mentioned outcomes. A family member or legal representative may answer all questions except those relating to HADS, IES-6, and TICS-m. Data on the EQ-5D-3L, visual analog scale, and Lawton and Brody scale from one month before the COVID-19 diagnosis, are collected retrospectively during the follow-up telephone calls. If information is missing regarding all-cause mortality, major cardiovascular events (cardiovascular death, non-fatal acute myocardial infarction, and non-fatal stroke), hospitalization within 12 months of study entry, or return to work or education, it may be evaluated retrospectively during any subsequent telephone call.

### Data quality procedures

Online standardized case report forms, available for smartphones, tablets, and personal computers, are used for data recording. Online data collection and management have several benefits including standardization, reliability, and data safety. All data are stored and managed with REDCap (Research Electronic Data Capture – https://www.redcapbrasil.com.br).^32^ The principal investigator assigns each investigator a unique non-transferable username and password to access the study platform. Specific permissions within the study platform are required.

### Data quality and safety

To ensure data quality and safety, the following procedures are followed:^33-35^ 1. All research staff undergo a training session on good clinical practices, study procedures, and data collection; 2. All investigators have access to the coordinating center to resolve any study issues; 3. All data management complies with the Brazilian General Data Protection Regulation (Law No. 13709 of August 14, 2018);^36^ 4. Database access is protected by a unique non-transferable username and password given to each study participant; 5. The dataset is automatically backed up every 24 hours. Data extraction for statistical analysis is performed with data anonymization, allowing for data consistency checking and remote monitoring of procedures; 6. The principal investigator periodically checks for data inconsistency. In case of errors, the investigators are notified and requested to correct the data entry; 7. Telephone calls are recorded and audited for data consistency. The audio files are stored with data anonymization on a server similar to that for clinical data. Access to audio files is granted by a unique username and password; 8. The coordinating center reviews detailed reports on patient screening, inclusion criteria, follow-up, data consistency, and data completeness monthly and takes immediate actions to resolve any issues; and 9. Statistical procedures are run throughout the study to identify potential data fraud.

### Sample size

An estimated sample size of 906 non-hospitalized patients with COVID-19 is needed to conduct a multiple linear regression with five predictors and a cross-correlation of 0.25 according to the PEAR method.^33^ Accounting for a 10% rate of lost to follow-up or withdrawal of consent, the final sample will include 997 participants. Given an anticipated hospitalization rate of 2-5% within 21 days, the sample size will be increased to 1047 participants for the primary analysis of non-hospitalized patients.

### Statistical analysis

Statistical analysis will be conducted once all data have been obtained, the dataset has been cleaned and locked, and the protocol has been submitted for publication. Firstly, a descriptive evaluation of the data will be conducted. Categorical variables will be presented as absolute and relative frequencies. Continuous variables will initially be assessed for data distribution using the Shapiro-Wilk test and visual inspection of the variables’ histograms. Continuous variables will be presented as either mean and standard deviation or median and interquartile ranges. The association between study outcomes and independent variables will be analyzed using Generalized Estimating Equations (GEE), both univariate and multivariate. Both unadjusted and adjusted values, along with 95% confidence intervals and p-values for each estimate, will be presented. Exploratory analyses will be performed with subgroup analyses on the outcomes. For continuous variables, either t-tests or Wilcoxon tests will be used depending on the data distribution, and for categorical variables, the chi-square test will be employed. Regression models will also be conducted depending on the type of the outcome of interest. A significance level of 0.05 and a 95% confidence interval will be considered for all statistical analyses.

The coordinating center will contact local investigators to correct inconsistent or missing data. If the data are still missing, no imputation will be performed for baseline data. Missing EQ-5D-3L assessments will be imputed using the last observation carried forward, except for deceased patients, who will receive a score of zero over the follow-up after the event. Missing values for the modified Barthel index, HADS, and IES-6 will be replaced by the mean of the answered items in the same subscale, if at least half of that subscale has been answered. Statistical analyses will be performed using R version 4.2.2 (R Foundation for Statistical Computing).

### Ethics and dissemination

#### Ethical approval and consent procedures

The study meets to the guidelines outlined in the Brazilian National Health Council Resolution 466 of December 12, 2012, the International Council for Harmonization guideline E6 addendum on good clinical practice (2nd revision), and the Brazilian General Data Protection Regulation (Law No. 13709 of August 14, 2018).^36^ The study was started only after approval of the protocol by the ethics committees of the institutions. Written or electronic informed consent is obtained from each eligible participant at the time of enrollment, and the language used is clear and inclusive, providing information on the objectives, methodology, data collection, and registration process of the study, in accordance with the Brazilian National Health Council Resolution 466/2012.

The local investigator reads the consent form to the screened participants or their legal representative, explaining the potential risks and benefits of the study. All participants are volunteers and can withdraw their consent at any time with no impact on their care. The investigator also informs the participants that their identification registry will be recorded and can be accessed by local health surveillance authorities and the coordinating center, without violating participants’ confidentiality. The consent form is registered with the current date and signed by both the participant or legal representative and the investigator only after the study procedures have been clarified and before any study protocol has been applied. One copy of the signed consent form is retained by the participant, and the other by the investigator.

According to the Circular Letter No. 2/2021 of the Brazilian National Research Ethics Committee, linked to the Brazilian Ministry of Health, issued on February 24, 2021, which provides for instructions on performing research and ethics committee activities in virtual environments during the COVID-19 pandemic, a digital signature can be accepted in centers where patients are followed up remotely or when the patients or their legal representative are unable to come to the center to consent. Regardless of the consent format, the investigator is responsible for holding proof of consent.

#### Dissemination

The investigators plan to present the study findings at medical meetings and conferences and to prepare a manuscript for publication in a peer-reviewed medical journal. The study’s steering committee will determine which results are to be published and to which medical journal they will be submitted. Authorship will be determined in accordance with the definition of the International Committee of Medical Journal Editors (ICMJE).

## Data sharing

The authors encourage third-party researchers to contact the corresponding author for data sharing and access to unpublished data. The use of a data-sharing application is under consideration by the study’s steering committee.

## Discussion and study update

Our study aims to investigate the impact of long COVID-19 on the quality of life and outcomes of patients who have had mild COVID-19. Most previous studies on symptom persistence have focused on patients with moderate-to-severe COVID-19 requiring hospitalization and experiencing a high burden of comorbidities.^6-8^ By focusing on patients with mild COVID-19, our study can identify those who are at risk of developing long-term symptoms and inform public health policies targeting specific subgroups of patients who are more likely to benefit from follow-up care.

A systematic review that analyzed nine studies of patients with mild COVID-19 and a follow-up of several weeks after infection showed that symptom persistence beyond 3 weeks ranged from 10% to 35%, with fatigue as the most common symptom. Other persistent symptoms after infection included dyspnea, cough, chest pain, headache, mental and cognitive decline, and taste and smell disorders. Additionally, the persistence of symptoms after SARS-CoV-2 infection was found to significantly impact work-related activities.^37^ However, the impact of symptoms lasting beyond three months remains unknown.

Population data from Brazil during the first year of the COVID-19 pandemic have shown an increase in cardiovascular mortality.^38^ This is concerning given that the SARS-CoV-2 virus can impact the cardiovascular system through several mechanisms, including microvascular dysfunction, oxygen supply-demand mismatch, direct myocardial injury, and cardiomyocyte toxicity during the acute phase of the disease.^14,39^These mechanisms may also contribute to long-term cardiovascular outcomes. Therefore, it is important to evaluate and follow-up patients for post-acute cardiovascular sequelae to identify those at risk and treat secondary cardiac diseases such as heart failure.^13,16,40^ Despite these efforts, the actual burden of long COVID on cardiovascular disease remains unknown and may require more time to be determined. The Post-COVID Brazil study 2 aims to shed light not only on the burden of cardiovascular symptoms in patients with COVID-19 but also on long-term outcomes.

The strength of this study lies in its prospective design, which includes a large number of patients with mild COVID-19 from various centers in Brazil, and centralized outcome adjudication with a 12-month follow-up. However, potential limitations include subjective interpretation of symptoms after COVID-19, which may be affected by medical visits and complementary tests conducted during the follow-up period. This self-assessment approach makes it difficult to determine whether the symptoms result from the SARS-CoV-2 infection or underlying comorbidities.

The study design and protocol were completed in December 2021. Patient recruitment was expected to be completed by December 2022, but screening will continue until the target population is achieved. As of the time of writing, one-third of the total number of participants have been enrolled in the study, with 27 active enrollment centers. The first phone calls to participants were made on February 24, 2022. The authors anticipate completing the 1-year follow-up of the study population by December 2023.
